# The virtual family conference in stroke rehabilitation: Education, preparation, and transition planning

**DOI:** 10.1177/02692155221146448

**Published:** 2022-12-27

**Authors:** Benjamin R Ritsma, Peter J Gariscsak, Aarti Vyas, Sophy Chan-Nguyen, Ramana Appireddy

**Affiliations:** 1 Department of Physical Medicine & Rehabilitation, 4257 Queen's University, Providence Care Hospital, Kingston, ON, Canada; 2 School of Medicine, 4257 Queen's University, Kingston, ON, Canada; 3 Department of Medicine–Division of Neurology, 4257 Queen's University, Kingston, ON, Canada

**Keywords:** Stroke rehabilitation, telemedicine, family conference, caregivers, health education

## Abstract

**Objective:**

To examine the virtual family conference as an educational, preparatory, and transition planning intervention in stroke rehabilitation.

**Design:**

Observational–cross-sectional study.

**Setting:**

Inpatient stroke rehabilitation.

**Subjects:**

Eighty-seven carers, participating in 48 conferences, were evaluated.

**Interventions:**

The virtual family conference, involving the patient, carer(s), and interdisciplinary rehabilitation team, completed prior to community transition. The conference protocol and framework, consisting of nine primary themes and additional sub-themes, are outlined. Teleconferencing was the utilized virtual modality.

**Main measures:**

Carers were assessed via questionnaires regarding pre- and post-conference rated: (1) stroke-related knowledge, (2) satisfaction with information provision, and (3) confidence, preparedness, and stress associated with community transition; by use of the Stroke Knowledge and Community Transition Preparedness Questionnaire, Mant *et al*. Information Satisfaction Questionnaire, and Kingston Caregiver Stress Scale.

**Results:**

Significant improvement in post-conference carer-rating was noted for knowledge, pertaining to stroke nature/impairments, stroke management/prevention, functional status, and community services. Significant gains were demonstrated in post-conference satisfaction with information provided regarding stroke and discharge planning, across all assessed topics. There was also a significant increase in carer-reported confidence and preparedness for the community transition as well as a significant reduction in self-perceived stress for elements of the caregiving role. Organization of community follow-up care was consistently enabled within the proposed framework.

**Conclusions:**

The virtual family conference intervention demonstrated efficacy in facilitating carer education and preparation, along with discharge planning prior to community transition from stroke rehabilitation. Thus, illustrating potential benefits of family conferences and feasibility of their virtual application in stroke rehabilitative care.

## Introduction

Stroke is an unfortunately common and heterogeneous condition, associated with significant neurological deficits, functional impairments, and challenges in community reintegration, experienced in part by both patients and their carers/caregivers.^1–3^ The significant contribution of carers to post-stroke recovery and community transition has been increasingly recognized,^3–6^ as have limitations in the facilitation of their preparation for this role.^3,7–9^ Moreover, the implementation of recent healthcare funding policies has generally shortened hospital length of stay, leaving carers with less time to prepare.^7,8^ The potential impact of the role on carer well-being has been noted,^3,7–11^ along with subsequent implications for patient response to rehabilitation.^3,8,12^ Carer and patient education and transition planning can contribute to enhanced satisfaction, improved outcomes, reduced stress, better quality of discharge, as well as improved quality of life,^3,8,12–15^ and as such, has been highlighted in international clinical guidelines.^4,13,16^ However, deficiencies in stroke health literacy^5,6,14,15^ and discharge planning^3,7–9^ are noted, along with the identified need for added research into means to address these issues.^3,5,14,15,17^

The family conference, a care meeting involving the patient, their carer(s), and an interdisciplinary care team, is utilized in several healthcare fields, such as rehabilitation, geriatric medicine, and palliative care.^1,11,18–20^ It is employed as a patient- and family-centered approach to enhance communication, provide updates, and help in discharge planning, treating the patient in the context of their support network.^1,11,18–20^ Nevertheless, there is a paucity of data pertaining to the impact of the family conference in stroke rehabilitation, as well as no guidelines and limited research related to the framework for their provision in this context.^1,6,18,21^ During the COVID-19 pandemic there has been a significant expansion in virtual care delivery,^22^ and at our center, the stroke rehabilitation team shifted to a virtual family conference, as a strategy to maintain carer education and engagement. To date, there is limited data exploring the virtual delivery of family conferences, including none within the setting of stroke rehabilitation. The objective of this study was to evaluate the utility of the virtual family conference as an educational, preparatory, and transition planning intervention during inpatient stroke rehabilitation, exploring the impact on carer-rated: (1) stroke/stroke recovery knowledge, (2) satisfaction with information provision, and (3) confidence, preparedness, and stress around community transition.

## Methods

The study sample was obtained from patients admitted to the inpatient stroke rehabilitation program at Providence Care Hospital, an academic rehabilitation hospital affiliated with Queen's University in Kingston, Ontario, Canada, from September 1, 2020, to April 8, 2021. The eligibility criteria included: (1) patient admitted post-stroke for stroke rehabilitation, with identified carer(s) (referring to family, close friends, or other individuals who comprise a patient's identified support network) to attend a proposed virtual family conference, (2) completed a virtual family conference, and (3) community transition planned, to a non-institutionalized care environment (i.e., non-long-term care/nursing home setting). All eligible patients and their identified carers were informed of the study and asked if they would like to be contacted with added information. If so, a non-clinical study member contacted the interested carers, provided study information, and obtained consent. Data was collected remotely using electronic chart review and telephone/online collection techniques, targeted within 1–2 days prior to and after the family conference based upon carer availability, employing REDCap.^23^ The study was approved by the Queen's University Health Sciences and Affiliated Teaching Hospitals Research Ethics Board and is outlined using the STROBE reporting guidelines.^24^

**Figure 1.**
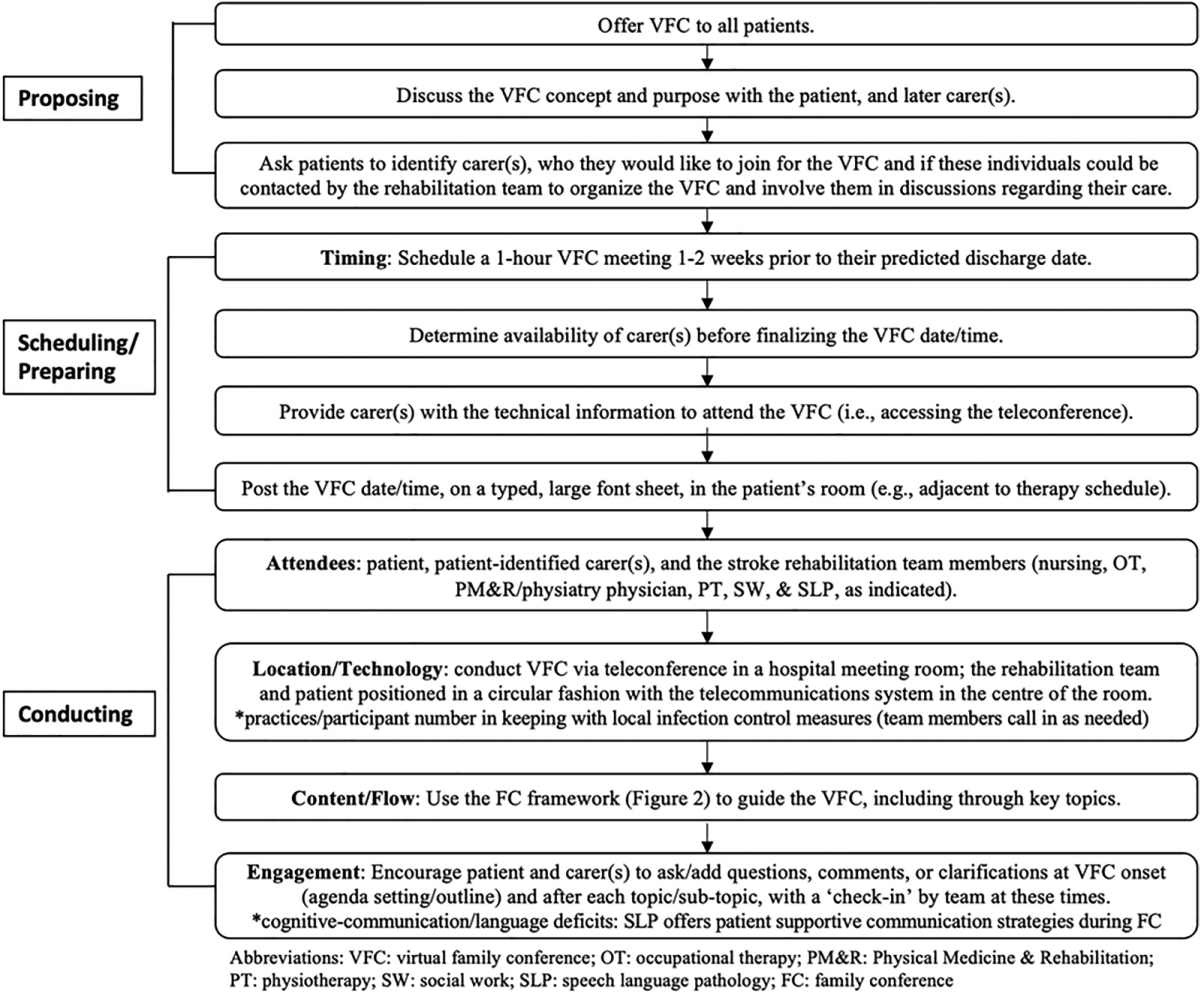
Virtual family conference protocol.

**Figure 2.**
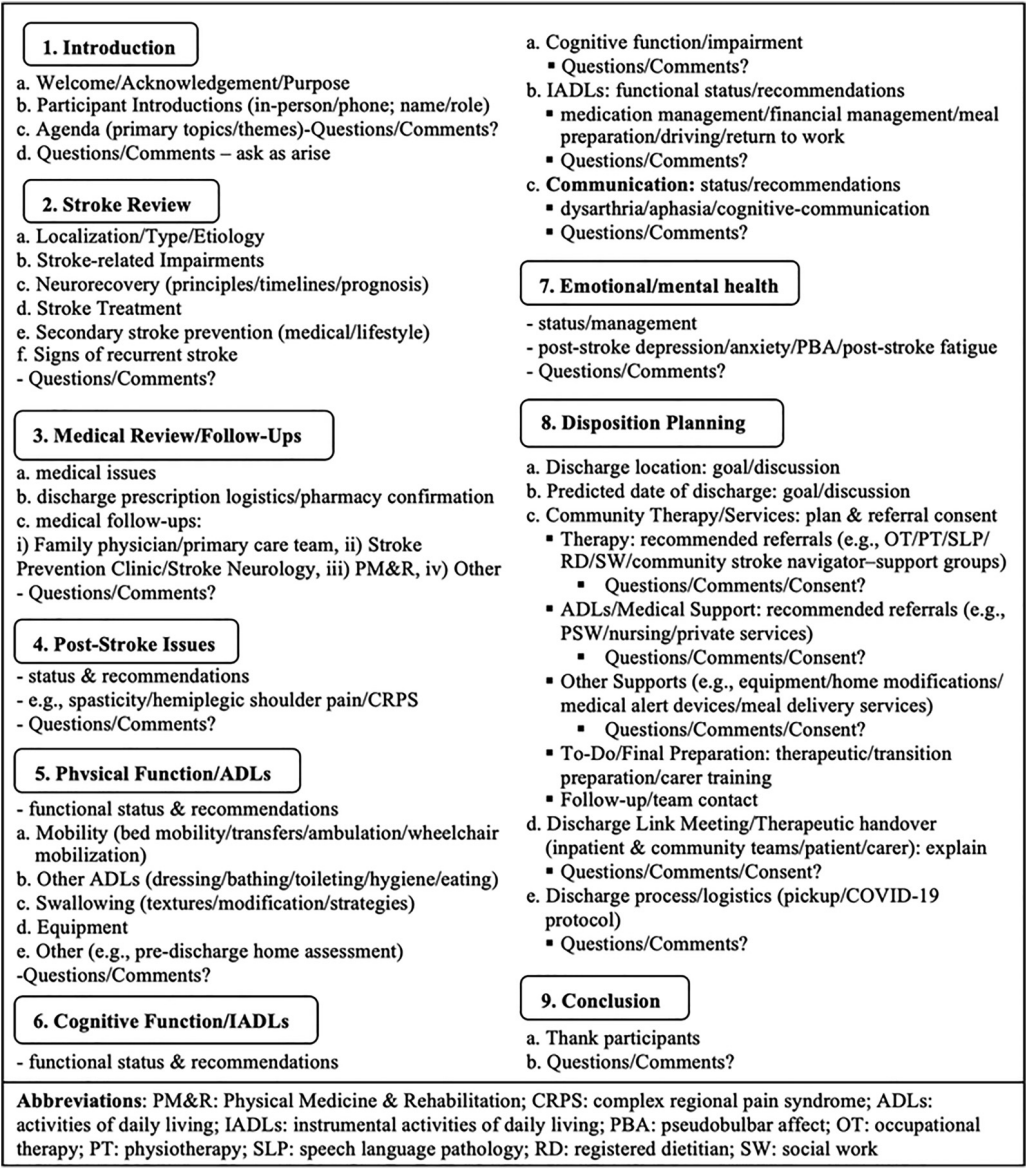
Family conference framework.

The virtual family conference intervention consists of multiple steps, for which a standard protocol was developed and utilized, as described (Figure 1). A framework (Figure 2) was also established by our stroke rehabilitation team to guide family conferences. Given the COVID-19 pandemic, it was further optimized for virtual delivery. It entails nine primary themes/topics, with additional sub-topics (Figure 2). The conversation for themes 1)–4) was led by the stroke rehabilitation team physiatry physician, with additions from other members as indicated. Topics 5)–9) also featured information provision from the interdisciplinary team (nursing, occupational therapy, physiatry, physiotherapy, social work, and speech language pathology, listed alphabetically). As noted (Figure 1), given the available institutional technology/technological support, along with the aim to maximize access and minimize required digital literacy for carers, we employed the teleconference as the single mode of virtual delivery.

The primary study objective was to assess, via a within-subject comparison, the impact of the virtual family conference intervention on carers, related to: (1) stroke-related knowledge, (2) satisfaction with information provision, and (3) community transition planning, namely associated carer confidence, preparedness, and stress. To do so, participating carers were asked to complete three online pre- and post-virtual family conference rating questionnaires, including: (1) Stroke Knowledge and Community Transition Preparedness Questionnaire, (2) Mant *et al*.^25^ Information Satisfaction Questionnaire, and (3) Kingston Caregiver Stress Scale.^26^

The Stroke Knowledge and Community Transition Preparedness Questionnaire (see Table 3A of Results) was utilized to assess carer-rated a) knowledge (patient/case-specific), related to Q1) stroke and post-stroke condition/impairments, Q2) stroke management/secondary prevention, Q3) functional status, Q4) recommended community services/supports, and b) confidence/preparedness for community transition, Q5). This questionnaire was designed for the purpose of this study and intended to represent five major educational themes featured in the virtual family conference intervention (Figure 1) and identified in the stroke literature.^1,4,6,13–16,27^ The Mant *et al*.^25^ Information Satisfaction Questionnaire (see Table 3B of Results), was employed to evaluate carer satisfaction with information provision related to stroke (e.g., what it is, causes, prevention, and nature) and post-stroke community services. It has previously been utilized in the assessment of the impact of another post-stroke information provision intervention, information packs, on carers.^25^ The Kingston Caregiver Stress Scale (see Table 3C of Results), namely four pertinent questions from its ‘care giving issues’ subscale, was used to gauge the level of carer-perceived stress regarding caregiving.^26^ It has been validated in the evaluation of the self-perceived levels of stress in caregivers, referring to community living lay carers, of those with cognitive impairment and disability, showing high test-retest reliability and validity.^26,28^

**Table 3.** Pre- and post-family conference carer-rating questionnaires.

	Pre-family conference	n (%)	Post-family conference	n (%)	p value
**A. Stroke Knowledge and Transition Preparedness Questionnaire**					
1) Overall, what would you say is your level of **knowledge** of your family member/friend's **stroke** (e.g., location/type/cause) and **post-stroke condition/impairments**?	Unaware	3 (3.45)	Unaware	0	.081
	Poor	7 (8.05)	Poor	2 (2.30)	.087
	Low	18 (20.69)	Low	2 (2.30)	<.001
	Mediocre	30 (34.48)	Mediocre	4 (4.60)	<.001
	Good	18 (20.69)	Good	44 (50.57)	<.001
	Excellent	11 (12.64)	Excellent	35 (40.23)	<.001
2) Overall, what would you say is your level of **knowledge** of the **stroke management and prevention** strategies for your family member/friend?	Unaware	12 (13.79)	Unaware	0	<.001
	Poor	13 (14.94)	Poor	1 (1.15)	<.001
	Low	18 (20.69)	Low	4 (4.60)	.001
	Mediocre	24 (27.59)	Mediocre	12 (13.79)	.025
	Good	16 (18.39)	Good	46 (52.87)	<.001
	Excellent	4 (4.60)	Excellent	24 (27.59)	<.001
3) Overall, what would you say is your level of **knowledge** of your family member/friend's **post-stroke function** (e.g., activities of daily living/ADLs & instrumental activities of daily living/IADLs)?	Unaware	6 (6.90)	Unaware	0	.013
	Poor	6 (6.90)	Poor	0	.013
	Low	23 (26.44)	Low	3 (3.45)	<.001
	Mediocre	20 (22.99)	Mediocre	14 (16.09)	.251
	Good	28 (32.18)	Good	36 (41.38)	.208
	Excellent	4 (4.60)	Excellent	34 (39.08)	<.001
4) Overall, what would you say is your level of **knowledge** of the **recommendations for services,** available to support your family member/friend, **post-discharge**?	Unaware	14 (16.09)	Unaware	0	<.001
	Poor	8 (9.20)	Poor	0	.004
	Low	15 (17.24)	Low	1 (1.15)	<.001
	Mediocre	11 (12.64)	Mediocre	9 (10.34)	.634
	Good	24 (27.59)	Good	39 (44.83)	.018
	Excellent	15 (17.24)	Excellent	38 (43.68)	<.001
5) Overall, how **confident/prepared** do you feel about the **plan** for your family member/friend's **transition** back home/to the **community**?	Unaware	8 (9.20)	Unaware	0	.004
	Poor	9 (10.34)	Poor	1 (1.15)	.014
	Low	15 (17.24)	Low	5 (5.75)	.018
	Mediocre	21 (24.14)	Mediocre	11 (12.64)	.050
	Good	22 (25.29)	Good	38 (43.68)	.011
	Excellent	12 (13.79)	Excellent	32 (36.78)	<.001
**B. Mant *et al*.** ^25^ **Information Satisfaction Questionnaire**					
Do you feel you know enough about what a stroke is?	Yes	63 (72.41)	Yes	81 (93.10)	<.001
	No	24 (27.59)	No	6 (6.90)	<.001
Would you like more information about the causes of stroke?	Yes	59 (67.82)	Yes	37 (42.53)	<.001
	No	28 (32.18)	No	50 (57.47)	<.001
Would you like more information about preventing another stroke?	Yes	64 (73.56)	Yes	48 (55.17)	.011
	No	23 (26.44)	No	39 (44.83)	.011
Do you feel you have all the information you want on the causes and nature of stroke?	Yes	46 (52.87)	Yes	65 (74.71)	.003
	No	41 (47.13)	No	22 (25.29)	.003
Do you feel you have all the information you need about allowances and services after your family member were discharged?	Yes	28 (32.94)	Yes	69 (79.31)	<.001
	No	57 (67.06)	No	18 (20.69)	<.001
**C. Kingston Caregiver Stress Scale**^26^ – Care Giving Issues					
Are you having feelings of being overwhelmed, overworked, and/or overburdened?	No Stress	26 (30.23)	No Stress	32 (36.78)	.362
	Some Stress	10 (11.63)	Some Stress	21 (24.14)	.032
	Moderate Stress	25 (29.07)	Moderate Stress	23 (26.44)	.699
	A lot of Stress	21 (24.42)	A lot of Stress	4 (4.60)	<.001
	Extreme Stress	4 (4.65)	Extreme Stress	7 (8.05)	.360
Do you have feelings of being confined or trapped by the responsibilities or demands of care giving?	No Stress	28 (32.18)	No Stress	38 (43.68)	.118
	Some Stress	30 (34.48)	Some Stress	25 (28.74)	.416
	Moderate Stress	19 (21.84)	Moderate Stress	12 (13.79)	.165
	A lot of Stress	6 (6.90)	A lot of Stress	11 (12.64)	.202
	Extreme Stress	4 (4.60)	Extreme Stress	1 (1.15)	.173
Do you ever have feelings related to a lack of confidence in your ability to provide care?	No Stress	35 (40.23)	No Stress	49 (56.32)	.034
	Some Stress	18 (20.69)	Some Stress	20 (22.99)	.714
	Moderate Stress	13 (14.94)	Moderate Stress	10 (11.49)	.502
	A lot of Stress	16 (18.39)	A lot of Stress	7 (8.05)	.044
	Extreme Stress	5 (5.75)	Extreme Stress	1 (1.15)	.096
Do you have concerns regarding the future care needs of your spouse/relative?	No Stress	16 (18.82)	No Stress	29 (34.12)	.024
	Some Stress	14 (16.47)	Some Stress	12 (14.12)	.670
	Moderate Stress	16 (18.82)	Moderate Stress	15 (17.65)	.843
	A lot of Stress	25 (29.41)	A lot of Stress	21 (24.71)	.490
	Extreme Stress	14 (16.47)	Extreme Stress	8 (9.41)	.170

Patient demographic, stroke, and rehabilitation/discharge outcome-related data pertinent to each conducted conference was collected via the electronic medical record. Patient characteristics were analyzed using descriptive statistics and computed in frequencies where possible. For carer questionnaire responses, answer option frequencies were compared from pre- and post-virtual family conference ratings using a two-proportion Z-test, level of significance set at p < 0.05. Analysis was completed using SPSS version 26.0 (SPSS Inc., Chicago, IL).

## Results

In total, the study included 48 virtual family conferences conducted for 48 patients receiving inpatient stroke rehabilitation care, following a confirmed new stroke. Patient baseline characteristics are outlined in Table 1. The average patient age was 75.0 years old (SD ± 11.6), with 54.2% (26) being male. On the Functional Independence Measure, mean rehabilitation admission and discharge total scores were 71.0 (SD ± 21.9) and 101.8 (SD ± 17.7), while cognitive subscale scores were 23.7 (SD ± 5.6) and 28.1 (SD ± 4.7), respectively. Scoring on the five items within the cognitive sub-domains of communication (comprehension and expression) and social cognition (social interaction, problem solving, and memory) is further outlined in Table 2, demonstrating mean functionalities of modified dependence to modified independence.^29^ Additional patient rehabilitation outcomes are outlined in Supplementary 1. Patient frequencies of ‘independent’ status in activities of daily living increased across all measures following inpatient rehabilitation. Patient frequencies of varying levels of functionality for instrumental activities of daily living were measured at baseline (pre-stroke) and at discharge from rehabilitation. Across all instrumental activities of daily living none of the proportions at discharge returned to similar frequencies of baseline function. All patients demonstrated improvement in independent status across various aspects of functional mobility.

**Table 1.** Baseline patient characteristics.

Baseline characteristics	n (%)
Age, mean (SD)	75.0 (11.6)
Gender	
Male	26 (54.2)
Female	22 (45.8)
Stroke hemisphere	
Right	25 (52.1)
Left	20 (41.7)
Bilateral	3 (6.3)
Stroke type	
Ischemic	39 (81.3)
Hemorrhagic	9 (18.8)
Baseline comorbidities	
Hypertension	41 (85.4)
Diabetes	13 (27.1)
Atrial fibrillation	11 (22.9)
Coronary artery disease	10 (20.8)
Prior stroke	6 (12.5)
Cancer	5 (10.4)
Chronic obstructive pulmonary disease	3 (6.3)
Ischemic stroke acute treatment	
Thrombolysis	9 (18.8)
Thrombectomy	5 (10.4)

**Table 2.** Patient rehabilitation and discharge outcomes.

**A. Rehabilitation outcomes**	
**Functional independence measure (FIM), mean (SD)^**a**^**	
**Admission FIM**	
Total (/126)	71.0 (21.9)
Motor-subscore (/91)	47.4 (19.5)
Cognitive-subscore (/35)	23.7 (5.6)
Communication items	
Comprehension (/7)	4.85 (1.39)
Expression (/7)	5.20 (1.71)
Social cognition items	
Social interaction (/7)	5.49 (1.71)
Problem solving (/7)	4.10 (1.24)
Memory (/7)	4.02 (1.23)
**Discharge FIM**	
Total (/126)	101.8 (17.7)
Motor-subscore (/91)	73.7 (16.0)
Cognitive-subscore (/35)	28.1 (4.7)
Communication items	
Comprehension (/7)	5.88 (1.08)
Expression (/7)	6.07 (1.39)
Social cognition items	
Social interaction (/7)	6.12 (0.75)
Problem solving (/7)	5.17 (1.20)
Memory (/7)	4.98 (1.29)
Change – Total	+ 30.8 (16.6)
**Length of Stay-Rehab (days), mean (SD)**	32.1 (14.8)
**B. Discharge outcomes**	
**Discharge destination**	**n (%)**
Home	46 (95.8)
Other (non-institutionalized care setting)^b^	2 (4.2)
**Discharge follow-up care**	**n (%)**
Occupational therapy	48 (100)
Physiotherapy	48 (100)
Nursing visit	45 (93.8)
Personal support worker	29 (60.4)
Speech language pathology	15 (31.3)

^a^ n = 42, not captured for patients in low-intensity stroke rehabilitation.

^b^ Non-long-term care/nursing home environment.

Subsequent to the 48 virtual family conferences, 46 patients (95.8%) transitioned to the home environment and 2 (4.2%) transitioned to a non-institutionalized care setting (i.e., non-long-term care/nursing home) (Table 2). Community-based follow-up care was organized for all 48 patients (100%) for occupational therapy and physiotherapy, 45 patients (93.8%) for a one-time nursing visit (featuring a medication review), 29 patients (60.4%) for personal support worker care, and 15 patients (31.3%) for speech language pathology (Table 2).

Eighty-seven participating carers were involved in the 48 virtual family conferences, an average of 1.8 carers per meeting. Carers were more often female (66.7%) and at community transition 41.0% were to be living with the patient. Participating carers’ relationship to the patient, in descending order of frequency, was as follows: child (45.8%), spouse/partner (27.7%), other family relation (15.7%, including sibling, parent, niece/nephew, relative by marriage), and friend (10.8%). The participating carers provided pre- and post-virtual family conference ratings (Table 3) as follows: (1) Stroke Knowledge and Transition Preparedness Questionnaire: significant improvement in carer-rated stroke knowledge and confidence/preparedness for community transition was seen in post-conference ratings, across all 5 topics; (2) Mant *et al*.^25^ Information Satisfaction Questionnaire: significant gains were noted in carer satisfaction with stroke and discharge planning-related information provision following the conferences, across all 5 items; and (3) Kingston Caregiver Stress Scale: significant improvement was seen in post-conference ratings within three of four questions regarding carer self-perceived stress in their caregiving role (i.e., sense of being overwhelmed/overburdened, lacking confidence, and having concerns).

## Discussion

This study provides evidence for the family conference as an educational, preparatory, and transition planning intervention following stroke. Post-family conference results demonstrated significant improvement in carer-rated stroke knowledge, satisfaction with information provision, as well as confidence/preparedness and stress around the community transition. The investigation also illustrates feasibility for the virtual delivery of family conferences in stroke as well as a conference protocol and framework for interdisciplinary rehabilitation team guidance.

The family conference offers potential benefits in facilitating communication and planning between a care team, patient, and carer(s), however there is a paucity of related data regarding its impact within specific clinical settings, namely stroke rehabilitation.^1,11,18,20^ Given their importance in stroke care and recovery,^3–6^ international stroke guidelines outline the value of carer education, yet the optimal means to achieve this is not clear and reference to the family conference is inconsistent.^4,13,16^

There are also no specific guidelines and limited research pertaining to the provision (e.g., method/framework, content, or timing) of family conferences in stroke.^1^ One can look to the wider stroke literature for preferred features of educational strategies which the family conference approach is well-positioned to incorporate. The utilization of interventions which are active, proactive, interactive, individualized (i.e., patient/case-specific), and interdisciplinary has been recommended,^4,13–16^ aspects intrinsic to the nature of the family conference. Moreover, it offers a unique opportunity to provide education on crucial topics to the patient and multiple carers at the same point in time,^11^ which could promote added, more unified, and contextual understanding. In their thorough review of the family conference literature, given the limited available research directly within stroke rehabilitation, Loupis *et al*.^1^ extrapolated from work in other fields to propose process-based features for the family conference post-stroke. This included communicating the purpose of the meeting to patients/carers, providing a written/verbal invitation for participation, scheduling at a mutually acceptable time, establishing the agenda, acknowledging time taken by patients/carers to attend, and utilizing a comfortable environment which facilitates effective communication and contact.^1,18,19^ All of these elements are incorporated within our conference framework, and our study sheds novel insight upon the ability of the virtual meeting environment to facilitate this. We also suggest, among others (Figure 1), formal inclusion of: 1) participant self-introductions, particularly relevant in the virtual context, and 2) encouragement of patient/carer input at meeting introduction and “check-ins” throughout, to stimulate dynamic engagement.

Related to conference content, the broader stroke education literature would suggest inclusion of key topics such as the nature of stroke, stroke management, outcome expectation, medical follow-up, community services, and discharge planning,^1,4,11,13–16,27^ as these reflect areas of deficiency. We have included these and added topics (Figure 2). In terms of timing, we planned the conference in relative proximity to the point of community transition (e.g., within 1–2 weeks of discharge date). In addition to meeting with and supporting/communicating with carers early and throughout an admission,^11,16^ there is some literature to suggest this timing may offer a more optimal juncture for providing accurate information regarding post-stroke prognostication, functional outcomes, support services, and practical community transition-based recommendations.^1,6,18,21^

The COVID-19 pandemic led to imposed restrictions on hospital visitor access and associated challenges in maintaining carer engagement in education and transition preparation. Thus, our stroke rehabilitation team shifted to the virtual provision of family conferences in March 2020, in line with public health measures and relevant guidance.^30^ In terms of virtual modality type, our model employed telephone/teleconferencing interactions, rather than video. This does remove the valuable element of visual communication, though requires the least technological literacy for users, our patients’ carers,^31^ and may reduce some of the technological challenges and barriers to access noted with more advanced modes.^32^ The feasibility of implementation and delivery of virtual care in stroke and other neurological diseases has been demonstrated.^33–35^ The patient-centered nature of virtual care is recognized^33,36^ and its application significantly expanded during the pandemic.^22^ Moreover, geography and the traditional in-person nature of the family conference, has been identified as a potential barrier to utilization^18^ and this comes with associated travel, time, and cost (e.g., transportation and missed work) considerations, which virtual strategies may help reduce.^32,33^ For instance, there may be added conference participation and reduced stress in doing so, if carers can engage in the setting that best aligns with their personal context. However, there is limited data exploring the virtual provision of the family conference. A pilot study in the critical care setting demonstrated no differences in satisfaction or decision-making between virtual and in-person family meetings.^37^ As the first study to investigate the virtual family conference in stroke rehabilitation, our analysis provides novel and positive insight on the feasibility and effectiveness of this alternative meeting environment in stroke care. We have also outlined a protocol and framework for doing so.

Limitations of this study include that it is a single-center design, with relatively small number of conferences, and a lack of true control arm. Given that the family conference was standard of care in our institute pre-pandemic, a control group without virtual conferences was not deemed to be an option. Moreover, the pandemic precluded our ability to use the in-person conference as a control. As such, we utilized a pre-post conference questionnaire model to act as an internal control. Finally, the authors acknowledge that while the results support the family conference as a valuable intervention, it represents one component of a larger education, communication, and collaborative planning approach to be employed throughout the stroke rehabilitation process.

There would be value in additional assessment of elements of the family conference following stroke and other conditions, including the optimal methods for delivery as well as the patient, carer, and healthcare professional perspective. The feasibility and significance of involving other healthcare providers (e.g., community therapists and primary care) merits exploration. Further inquiry into the impact on patient and carer-specific outcomes in the short and long term would be meaningful, including relative to various carer roles. Future studies assessing resource utilization, value, and feasibility of employing other virtual modalities as well as exploration of virtual compared to in-person conferences could be beneficial. In turn, this could bring forth added indication for the consistent incorporation of the family conference intervention within international stroke guidelines. Going forward, we anticipate and support the important role that virtual modalities will assume in stroke rehabilitative care, including education and transition planning strategies such as the virtual family conference. At a stage of less acute public health concerns, we recommend a patient and carer-directed approach in selecting the mode of family conference interaction (i.e., in-person, virtual, or hybrid combination).

**Clinical messages**

- Interdisciplinary stroke rehabilitation teams should look to assess and further develop their local family conference protocols, including for virtual delivery, as needed.
- Interdisciplinary stroke rehabilitation teams could consistently offer the family conference, as a means to facilitate patient and carer education and preparation for community transition.
- Virtual delivery of the family conference could be employed to alleviate barriers to participation (e.g., public health measures, travel, time, work, and out of pocket expenses), thus improving access and enabling the engagement of a broader patient support network in the stroke recovery process.

## Supplemental Material

sj-docx-1-cre-10.1177_02692155221146448 - Supplemental material for The virtual family conference in stroke rehabilitation: Education, preparation, and transition planning Click here for additional data file. Supplemental material, sj-docx-1-cre-10.1177_02692155221146448 for The virtual family conference in stroke rehabilitation: Education, preparation, and transition planning by Benjamin R Ritsma, Peter J Gariscsak, Aarti Vyas, Sophy Chan-Nguyen and Ramana Appireddy in Clinical Rehabilitation
