# Supplementary material for: The virtual family conference in stroke rehabilitation: Education, preparation, and transition planning
Source: Clin Rehabil. 2022 Dec 27;37(8):1099–110. doi: 10.1177/02692155221146448 (PMC10291377; doi:10.1177/02692155221146448)
Supplement: sj-docx-1-cre-10.1177_02692155221146448 - Supplemental material for The virtual family conference in stroke rehabilitation: Education, preparation, and transition planning [file sj-docx-1-cre-10.1177_02692155221146448.docx]

**Supplementary Material**

**Supplementary 1. Additional patient rehabilitation outcomes.**

| **Activities of Daily Living (ADLs)** | | **Admission**  n (%) | **Discharge**  n (%) |  |  | **Admission** n (%) | **Discharge**  n (%) |
| --- | --- | --- | --- | --- | --- | --- | --- |
| Feeding | Independent  Supervision  Assist  Dependent  Missing | 26 (54.2)  6 (12.5)  6 (12.5)  1 (2.1)  9 (18.8) | 37 (77.1)  2 (4.2)  6 (12.5)  1 (2.1)  2 (4.2) | Toileting | Independent  Supervision  Assist  Dependent  Missing | 16 (33.3)  5 (10.4)  16 (33.3)  3 (6.3)  8 (16.7) | 33 (68.8)  2 (4.2)  9 (18.8)  1 (2.1)  3 (6.3) |
| Grooming | Independent  Supervision  Assist  Dependent  Missing | 18 (37.5)  6 (12.5)  15 (31.3)  0  9 (18.8) | 34 (70.0)  4 (8.3)  8 (16.7)  1 (2.1)  1 (2.1) | Bladder Control | Independent  Supervision  Assist  Dependent  Missing | 19 (39.6)  0  1 (2.1)  3 (6.3)  25 (52.1) | 28 (58.3)  0  3 (6.3)  2 (4.2)  15 (31.3) |
| Bathing | Independent  Supervision  Assist  Dependent  Missing | 10 (20.8)  4 (8.3)  23 (47.9)  2 (4.2)  9 (18.8) | 18 (37.5)  9 (18.8)  19 (39.6)  1 (2.1)  1 (2.1) | Bowel Control | Independent  Supervision  Assist  Dependent  Missing | 18 (37.5)  1 (2.1)  1 (2.1)  3 (6.3)  25 (52.1) | 29 (60.4)  0  2 (4.2)  1 (2.1)  16 (33.3) |
| Dressing | Independent  Supervision  Assist  Dependent  Missing | 16 (33.3)  5 (10.4)  17 (35.4)  2 (4.2)  8 (16.7) | 28 (58.3)  2 (4.2)  16 (33.3)  1 (2.1)  1 (2.1) |  |  |  |  |
| **Instrumental Activities of**  **Daily Living (IADLs)** | | **Baseline**  n (%) | **Discharge** n (%) |  |  | **Baseline**  n (%) | **Discharge**  n (%) |
| Meal Preparation | Independent  Supervision  Assist  Dependent  Missing | 23 (47.9)  0  15 (31.3)  8 (16.7)  2 (4.2) | 11 (22.9)  11 (22.9)  14 (29.2)  8 (16.7)  4 (8.3) | Medical Management | Independent  Supervision  Assist  Dependent  Missing | 35 (72.9)  0  4 (8.3)  6 (12.5)  3 (6.3) | 11 (22.9)  16 (33.3)  9 (18.8)  9 (18.8)  3 (6.3) |
| Laundry | Independent  Supervision  Assist  Dependent  Missing | 22 (45.8)  0  11 (22.9)  10 (20.8)  5 (10.4) | 4 (8.3)  3 (6.3)  9 (18.8)  10 (20.8)  22 (45.8) | Financial Management | Independent  Supervision  Assist  Dependent  Missing | 21 (43.8)  2 (4.2)  10 (20.8)  11 (22.9)  4 (8.3) | 3 (6.3)  12 (25.0)  15 (31.3)  11 (29.2)  4 (8.3) |
| Cleaning | Independent  Supervision  Assist  Dependent  Missing | 19 (39.6)  0  11 (22.9)  11 (22.9)  7 (14.6) | 0  1 (2.1)  9 (18.8)  12 (25.0)  26 (54.2) | Outdoor Maintenance | Independent  Supervision  Assist  Dependent  Missing | 13 (27.1)  0  4 (8.3)  17 (35.4)  14 (29.2) | 0  1 (2.1)  2 (4.2)  17 (35.4)  28 (58.3) |
| **Functional Mobility** | | **Admission**  n (%) | **Discharge** n (%) |  |  | **Admission**  n (%) | **Discharge**  n (%) |
| Static Sitting Balance | Independent  Supervision  Assist  Dependent  Missing | 31 (64.6)  0  2 (4.2)  11 (22.9)  4 (8.3) | 27 (56.3)  0  1 (2.1)  0  21 (41.7) | Bed Mobility | Independent  Supervision  Assist  Dependent  Missing | 25 (52.1)  2 (4.2)  17 (35.4)  0  4 (8.3) | 41 (85.4)  0  2 (4.2)  2 (4.2)  3 (6.3) |
| Dynamic Sitting Balance | Independent  Supervision  Assist  Dependent  Missing | 24 (50.0)  1 (2.1)  2 (4.2)  17 (35.4)  4 (8.3) | 23 (47.9)  4 (8.3)  1 (2.1)  0  20 (41.7) | Transfers | Independent  Supervision  Assist  Dependent  Missing | 13 (27.1)  3 (6.3)  24 (50.0)  4 (8.3)  4 (8.3) | 35 (72.9)  5 (10.4)  5 (10.4)  0  3 (6.3) |
| Static Standing Balance | Independent  Supervision  Assist  Dependent  Missing | 15 (31.3)  0  3 (6.3)  24 (50.0)  6 (12.5) | 22 (45.8)  3 (6.3)  0  1 (2.1)  22 (45.8) | Ambulation | Independent  Supervision  Assist  Dependent  Missing | 5 (10.4)  8 (16.7)  18 (37.5)  13 (27.1)  4 (8.3) | 31 (66.0)  9 (18.8)  3 (6.3)  4 (8.3)  1 (2.1) |
| **Mobility Tests** |  |  |  |  |  |  |  |
|  | **Baseline**  Mean (SD) | **Discharge**  Mean (SD) |  |  | **Baseline**  Mean (SD) | **Discharge**  Mean (SD) |  |
| Berg Balance Scale (/56) | 27.7 (15.8) | 44.3 (10.7) | Ambulatory Endurance (m) | | 98.5 (106.7) | 316.9 (263.8) |  |
| Ambulatory Pace (m/s) | 0.44 (0.26) | 0.78 (0.34) |  |  |  |  |  |
